# Be Kind to Children

**Published:** 1880-03

**Authors:** 


### BE KIND TO CHILDREN.

**Reader, be kind to children—then your name will be held by them, in after years, with a grateful remembrance—for impressions formed in childhood, though trifling in their nature, are almost always indelible.**

**If we speak an unkind word to a child, how soon a shade of gloom will steal over its little brow: and if they have really done something that deserves censure, remember they are but children, and you must expect they will do things inconsiderately; but it is your duty to forgive, and treat them with kindness, for we are satisfied, from what has come under our own observation, that kindness will control an obstinate child far better than severity. If that be true, be kind to children.**

But perhaps a mother will say, "My child is so obstinate that I can not help speaking to it harshly." But remember, mother, your harsh words have lost their power, and if you would control your child, let your voice be low and soft, as the Æolian harp.

